# Retraction Note: Synthesis and characterization of ZrFe_2_O_4_@SiO_2_@Ade-Pd as a novel, recyclable, green, and versatile catalyst for Buchwald–Hartwig and Suzuki–Miyaura cross-coupling reactions

**DOI:** 10.1038/s41598-024-62395-3

**Published:** 2024-05-21

**Authors:** Mohamed J. Saadh, Hussam Elddin Nabieh Khasawneh, Geovanny Genaro Reivan Ortiz, Muhammad Ahsan, Dinesh Kumar Sain, Kareem Yusuf, Mika Sillanää, Amjad Iqbal, Reza Akhavan-Sigari

**Affiliations:** 1 Faculty of Pharmacy, Middle East University, Amman, 11831 Jordan; 2 Applied Science Research Center, Applied Science Private University, Amman, Jordan; 3 Chemical Engineering Department, Al-Balqa Applied University, Salt, Jordan; 4 Laboratory of Basic Psychology, Behavioral Analysis and Programmatic Development (PAD-LAB), Catholic University of Cuenca, Cuenca, Ecuador; 5 Department of Measurements and Control Systems, Silesian University of Technology, 44-100 Gliwice, Poland; 6 Department of Chemistry, Faculty of Science, S.P. College Sirohi, Sirohi, Rajasthan 307001 India; 7 Department of Chemistry, College of Science, King Saud University, P. O. Box 2455, Riyadh, 11451 Saudi Arabia; 8 Department of Biological and Chemical Engineering, Aarhus University, Nørrebrogade 44, 8000 Aarhus, Denmark; 9 Joint Doctoral School, Silesian University of Technology, 44-100 Gliwice, Poland; 10 Department of Health Care Management and Clinical Research, Collegium Humanum Warsaw Management University, Warsaw, Poland; 11 Department of Neurosurgery, University Medical Center Tuebingen, Tuebingen, Germany

Retraction of: *Scientific Reports* 10.1038/s41598-023-37680-2, published online 07 September 2023

The Editors have retracted this article.

After publication, concerns about the FTIR data in Figure 1 and XRD spectrum in Figure 2 were raised to the Editors. The Editors requested full raw data from the Authors, but the Authors did not provide a response, and the published Author Correction^1^ also does not address the concerns satisfactorily. The Editors have therefore lost confidence in the reliability of the article's findings and conclusions.

Geovanny Genaro Reivan Ortiz agrees with this retraction. The remaining authors did not respond to correspondence from the publisher about this retraction.
